# Increased household transmission and immune escape of the SARS-CoV-2 Omicron compared to Delta variants

**DOI:** 10.1038/s41467-022-33233-9

**Published:** 2022-09-29

**Authors:** Neda Jalali, Hilde K. Brustad, Arnoldo Frigessi, Emily A. MacDonald, Hinta Meijerink, Siri L. Feruglio, Karin M. Nygård, Gunnar Rø, Elisabeth H. Madslien, Birgitte Freiesleben de Blasio

**Affiliations:** 1 Norwegian Institute of Public Health, Oslo, Norway; 2 Oslo Centre for Biostatistics and Epidemiology, University of Oslo, Oslo, Norway; 3 Oslo Centre for Biostatistics and Epidemiology, Oslo University Hospital, Oslo, Norway

**Keywords:** Viral infection, Epidemiology, SARS-CoV-2

## Abstract

Understanding the epidemic growth of the novel SARS-CoV-2 Omicron variant is critical for public health. We compared the ten-day secondary attack rate (SAR) of the Omicron and Delta variants in households using Norwegian contact tracing data, December 2021 - January 2022. Omicron SAR was higher than Delta, with a relative risk (RR) of 1.41 (95% CI 1.27-1.56). We observed increased susceptibility to Omicron infection in household contacts compared to Delta, independent of contacts’ vaccination status. Among three-dose vaccinated contacts, the mean SAR was lower for both variants. We found increased Omicron transmissibility from primary cases to contacts in all vaccination groups, except 1-dose vaccinated, compared to Delta. Omicron SAR of three-dose vaccinated primary cases was high, 46% vs 11 % for Delta. In conclusion, three-dose vaccinated primary cases with Omicron infection can efficiently spread in households, while three-dose vaccinated contacts have a lower risk of being infected by Delta and Omicron.

## Abstract (web summary)

In this study the authors investigate household transmission of SARS-CoV-2 in Norway. They find that the secondary attack rate was higher for Omicron than Delta, but that among three-dose vaccinated contacts the secondary attack rate was lower for both variants compared to contacts with two doses.

## Introduction

By the end of 2021, the rapid global spread of the novel SARS-CoV-2 Omicron variant of concern (VoC) (Pangolin designation B.1.1.529 BA.1) caused major concern and an urgent need for knowledge about its transmissibility, the severity of disease and ability to escape vaccine immunity^1,2^. In Norway, the first SARS-CoV-2 Omicron outbreak was detected on 30 November at a time when the Delta variant was dominating^3^. At the same time, a large Omicron outbreak was detected after a Christmas party, causing an attack rate of 74% among participants, of which most (98%) were vaccinated with two doses^3^. Over the next 4–6 weeks, Omicron rapidly took over for the Delta variant in Norway. By week 2 in 2022, the Omicron variant was detected in >90% of the weekly national samples screened or sequenced for virus variants^4^. Early studies report that Omicron might have higher transmissibility than Delta^5–7^, although infection seems to cause less severe disease and lower risk of hospitalization^1,8^. The Omicron variant’s ability to escape vaccine immunity^9–11^ is likely an important contributor to the current rapid spread of the disease^7^. A highly immune evasive VoC could potentially challenge global control strategies. Thus, timely and relevant knowledge about transmissibility and risk of infection concerning the vaccination status of the population is of particular importance to guide health authorities.

Here, we use contact tracing data collected by Norwegian municipalities to estimate and compare household 10-day secondary attack rate (SAR) for the Omicron and Delta variants at a time when both variants were circulating throughout the country.

## Results

### Overall findings

In total, 1122 primary cases with confirmed Delta (41%) or Omicron (59%) and 2169 household contacts (60% for Omicron primary cases and 40% for Delta primary cases) were registered in the contact tracing system and included in the final data set. The analyses include data from 57 of the 365 municipalities in Norway, representing 8 of the 11 counties in the country. Characteristics of the cases and contacts are presented in Supplementary Methods, Section S3. We calculated the household 10-day SAR, the probability that a contact of a primary case was tested positive within ten days of his/her primary case’s test date. The overall SAR of households with Omicron was estimated at 51% (CI_95_: 48–54) compared to 36% (CI_95_: 33–40) with Delta, giving a significantly higher risk of infection in households with Omicron relative to Delta (Table 1). A primary case infected with Omicron had a significantly higher risk of COVID-19 transmission in both age groups than a primary case infected by Delta.

**Table 1:** Ten-day secondary attack rate (SAR) of Omicron and Delta stratified by age and vaccination status

Stratification	SAR (95% CI)		RR (95% CI)	Number of contacts	
	Omicron	Delta	Omicron vs Delta (Delta is ref.)	Omicron	Delta
Overall	0.51 (0.48−0.54)	0.36 (0.33−0.40)	1.41 (1.27−1.56)	1299	870
Age of contacts					
<16 years	0.58 (0.53−0.63)	0.42 (0.36–0.48)	1.38 (1.18−1.64)	356	278
16+ years	0.49 (0.45−0.51)	0.33 (0.30−0.37)	1.44 (1.27−1.65)	943	583
Age of primary cases					
<16 years	0.53 (0.48−0.58)	0.35 (0.30–0.40)	1.53 (1.29−1.82)	354	353
16+ years	0.50 (0.47−0.53)	0.37 (0.33−0.41)	1.35 (1.19−1.54)	945	517
Vaccine status, contacts					
Unvaccinated	0.59 (0.53−0.64)	0.47 (0.41−0.52)	1.27 (1.09−1.48)	322	297
1-dose vaccine	0.57 (0.48−0.66)	0.34 (0.23−0.46)	1.69 (1.18−2.55)	121	62
two-dose vaccine	0.50 (0.46−0.54)	0.32 (0.28−0.37)	1.55 (1.33−1.82)	654	445
three-dose vaccine	0.38 (0.31−0.44)	0.20 (0.11−0.30)	1.91 (1.19−3.40)	202	66
Vaccine status, primary cases					
Unvaccinated	0.57 (0.51−0.62)	0.38 (0.33−0.42)	1.51 (1.30−1.77)	329	420
1-dose vaccine	0.42 (0.34−0.49)	0.39 (0.29−0.51)	1.05 (0.76−1.52)	154	71
two-dose vaccine	0.51 (0.47−0.55)	0.35 (0.31−0.40)	1.44 (1.24−1.70)	713	360
three-dose vaccine	0.46 (0.36−0.55)	0.11 (0.02−0.29)	4.34 (1.52−25.16)	103	19

*SAR* secondary attack rate, *RR* relative risk, unadjusted for covariates, *CI* confidence interval.

### Vaccination effect

The risk of infection in all vaccination groups of contacts was significantly higher in households with Omicron relative to Delta (Table 1).

Generally, the SAR in households with three-dose vaccinated primary cases and contacts was lower than in households with unvaccinated primary cases and contacts. Primary cases who had three doses of vaccine and were infected with the Omicron variant were found to have a considerably higher risk (RR: 4.34; CI95: 1.52–25.16) of transmitting SARS-CoV-2 to their household compared with three-dose vaccinated primary cases infected with the Delta variant. A similar trend was observed when the primary case was unvaccinated or had only two doses of vaccine, although the relative risks were lower (Unvaccinated: RR: 1.51; CI_95_: 1.30–1.77, two-dose vaccination: RR: 1.44; CI_95_: 1.24–1.70).

Vaccine efficacy (VE) for adult contacts in the three-dose vaccination group was lower for Omicron (45%; CI_95_: 26–57) compared to Delta (65%; CI_95_: 42–80) and was higher than the VE among contacts with two doses of vaccination (Table 2). In the latter group, we found the protection against infection with Omicron to be 27% (CI_95_: 6–49), whereas it was equal to 42% (CI_95_: 23–55) for Delta.

**Table 2:** Vaccine efficacy (VE) of Omicron and Delta by contact vaccination status (16+ years)

Vaccine status	VE % (95% CI)	
	Omicron	Delta
Unvaccinated	Ref.	Ref.
1-dose vaccine	22 (0–46)	31 (0−72)
two-dose vaccine	27 (6−49)	42 (23−55)
three-dose vaccine	45 (26−57)	65 (42−80)

*VE* vaccine efficacy, *CI* confidence interval.

Two-dose vaccinated primary cases had a similar risk as unvaccinated primary cases in the transmission of Omicron infection to their adult household members (RR: 1.04; CI_95_: 0.79−1.49), Table 3. The same pattern was observed for the primary cases with three vaccine doses versus unvaccinated primary cases (RR: 0.99; CI_95_: 0.68−1.49). In contrast, three-dose vaccinated Delta primary cases had an 80% lower risk of Delta transmission (RR: 0.18; CI_95_: 0.01−0.70) relative to the unvaccinated primary cases. When estimating the risk of infection, stratified by age groups, gender, and the time since the last vaccine dose (in two-dose vaccinated contacts), we found no significant differences (Table 3).

**Table 3:** Relative risk of Omicron and Delta infection stratified by different covariates

RR within variant	RR (95% CI)	
	Omicron	Delta
Primary case vaccination status (16+)		
Unvaccinated	Ref.	Ref.
1-dose vaccine	1.01 (0.64−1.58)	0.99 (0.51−1.64)
two-dose vaccine	1.04 (0.79−1.49)	0.63 (0.46−0.89**)**
three-dose vaccine	0.99 (0.68−1.49)	0.18 (0.01−0.70)
Age of contacts		
<16 years	1.28 (0.97−1.83)	1.35 (0.83−2.60)
16–44 years	1.16 (0.87−1.65)	1.05 (0.65−2.03)
45–64 years	0.97 (0.73−1.39)	1.14 (0.69−2.23)
65+ years	Ref.	Ref.
Gender of contacts, 16+ years		
Female	Ref.	Ref.
Male	0.90 (0.79−1.03)	1.03 (0.82−1.29)
Time since last vaccination, two-dose vacc. contacts 16+ years		
≤6 months	0.89 (0.60−1.19)	0.65 (0.34−1.07)
>6 months	Ref.	Ref.

*RR* relative risk, unadjusted for covariates, *CI* confidence interval.

We conducted a sensitivity analysis using five days between the infected contacts’ test dates and their primary cases’ test dates instead of ten days and calculated the so-called SAR5. Results are provided in the Supplementary Methods, Section S4, and show the same disease transmissibility and vaccination effectiveness pattern for Omicron and Delta.

## Discussion

During a period when both the Omicron and Delta were circulating, we found a higher overall household 10-day SAR for the Omicron (51%) compared to the Delta (36%) variant. This finding aligns with observations from studies of individual-level registry data in Norway^12^ and Denmark^7^, and the UK^13^. The SAR estimates were generally higher in our study, which could be due to various reasons such as differences in the testing regimes or discrepancies in the capacity and procedures for registering household contacts. In this study, we have used contact tracing data, which may give higher estimates of SAR than registry-based studies since exposure is verified through personal interviews. Furthermore, since the included children <16 years mainly were unvaccinated or with one dose of vaccination, this could contribute to the overall higher household SAR for both Delta and Omicron observed in our study. The overall SAR was substantially higher in households infected with Omicron, in line with the rapid takeover of Omicron from Delta in Norway. However, household exposure is often prolonged and repeated compared to social contacts in society, and preferably a complete evaluation should consider all close contacts. Unfortunately, the inclusion of non-household close contacts was not possible due to variations in contact tracing practices during the study period and between localities.

Unlike Lyngse et al.^7^, our study finds a significantly higher SAR for Omicron than Delta among unvaccinated household members, suggesting that intrinsic transmissibility is higher for the Omicron variant. This finding supports early assumptions that the Omicron is fundamentally more transmissible than Delta^14^. We also found a higher household SAR among two-dose and three-dose vaccinated household contacts exposed to Omicron primary cases compared to households exposed to Delta primary cases, indicating that immune evasion contributes to the increased transmissibility of the Omicron variant. The group of one-dose individuals is heterogeneous and with varying vaccination dates, which may explain the lower RR of Omicron infection versus Delta compared to the other vaccination groups. Also, adolescents aged 12–19 years were overrepresented in this group. However, because we did not adjust for age in this analysis, our results should be interpreted cautiously.

Vaccination with two and three doses seemed to give lower protection against infection for Omicron than Delta, which is supported by other epidemiological studies and neutralization studies, likely related to a large number of mutations in the spike (S) protein compared to Delta^9^. While the protective effect of three-dose vaccination against Omicron infection (VE) was significant, its protection to prevent onward transmission from cases seems rather low compared to Delta. Our results indicate that three-dose vaccinated primary cases had approximately a fourfold higher risk of transmitting Omicron to their household contacts relative to Delta primary cases, which is higher than the risk of Omicron transmission versus Delta in two-dose and unvaccinated primary cases. These results have not been investigated in a previous study in Norway^12^. However, we did not adjust for age and time since vaccination, and thus, our results should be interpreted with caution.

Since exposure within the same household is often prolonged over several days, the estimated SAR might reflect several transmission chains. We performed a sensitivity analysis, reducing the exposure window to 5 days. The SAR5 was shown to give comparable results, thus, corroborating the main findings.

We assumed that the test sensitivity did not vary between Omicron and Delta. PCR was frequently used to identify SARS-CoV-2 infection, accounting for 80–83% of the primary cases and 86–87% of the positive contacts. The remaining cases were detected with an antigen test and PCR except for eight contacts recorded exclusively with an antigen test. In Norway, all positive-result antigen tests registered in the national health registries have been performed by trained health personnel at the official test centers. Only antigen tests procured by the government have been in use. The analytical sensitivity of the employed antigen tests to detect Omicron and Delta were found to be comparable in an assessment done at the Norwegian Institute of Public Health (data not published). This finding is supported by international studies^15,16^. A strength of our study is that contact tracing data, in contrast to pure registry-based data, provides qualitative data on true exposures identified through personal interviews. Ideally, contact tracing data should be combined with either contact category, e.g. household contact, friend, colleague etc., or updated information on place of living, to separate household contacts from other contacts. We matched contact tracing data with household registry information to identify which contacts belonged to the same household. Since we did not have access to either contact category or current living address, the actual number of household contacts might diverge from our estimates.

Our study has several limitations. Municipalities have used different approaches to contact tracing and testing of contacts based on local priorities, resources, and regulations. This means that the quality of the contact tracing data will likely vary between municipalities and over time. Therefore, we have restricted our analyses to household contacts, as the contact tracing teams have generally prioritized them. However, we cannot rule out that some household contacts have escaped registration in the contact tracing system, particularly in periods of very high incidence. Municipalities were recommended to prioritize contact tracing around cases with a high risk of severe illness or high risk of transmission to many contacts in situations with particularly high incidence.

We assumed that household contacts were tested at least once during their ten days of quarantine, especially since a negative test result on day seven would allow termination of quarantine. However, since we did not include the test record of the contacts (including any positive result of antigen-self tests not confirmed in the national surveillance system), we cannot rule out that the true number of infected contacts might be higher than captured in this study. Still, the national regulation required all positive-result home tests to be confirmed by a PCR test to obtain an immunity certificate. For this reason, most positive test results are likely registered. Further studies are needed to assess the protective effect of vaccination against the disease. Unfortunately, our data did not contain information about symptoms. Also, we did not include detailed information on the time since the last vaccination or vaccine types administered in our analyses. These characteristics should be more thoroughly investigated in separate studies focusing on VE. In conclusion, our study indicates that the higher overall SAR among household contacts of Omicron cases is most likely due to higher intrinsic transmissibility of this variant and lower vaccine effectiveness. As reported by others, contacts vaccinated with three doses had a lower risk of infection with Delta and Omicron, but our findings suggest that three-dose vaccinated cases with Omicron infection can spread effectively in households.

## Methods

The study complies with all relevant ethical regulations in accordance with the Norwegian Health Preparedness Act, paragraph 2–4.

### Study design

We conducted a registry-based cohort study using data from the Norwegian COVID-19 pandemic preparedness register, Beredt C19^17^. Beredt C19 receives individual-level information from Norwegian health registries, which can be linked using unique personal identification numbers. The preparedness register aims to enable rapid knowledge generation on the spread of COVID-19 to support national authorities in crisis management and preparedness planning. Beredt C19 collects information from various national registries, including details on Norwegian residents who have tested positive for SARS-CoV-2, date of testing, variant detection, vaccination record, and demographic characteristics, including age, gender, and place of residence. Furthermore, the register receives digital contact tracing data on a voluntary basis from 65/365 Norwegian municipalities, enabling the linkage of index cases to their traced contacts. The contact tracing data is based on personal phone interviews conducted by trained health personnel from the municipal health services. Detailed information on the specific Beredt C19 data sources that were used in this study is shown in Supplementary Methods, Section S1.

### Study population

The study population was households registered in the municipal contact tracing system of 64 municipalities within the study period. We defined a primary case as the person in the household registered as an index case in the KS Fiks Smittesporing system and tested positive for either the SARS-CoV-2 Omicron or Delta variant during the study period, but not listed as a contact in the system. This primary case is then the person who initiated the contact tracing in that household. Household contacts of the primary cases were identified by matching household identification numbers of the primary case and contacts.

### Test methods

In Norway, registered positive cases are based on testing by trained health personnel at local test centers using PCR or rapid antigen tests. All primary cases were detected with PCR (Delta: 80%; Omicron 81%) or an antigen test and PCR (Delta: 20%; Omicron 19%). Among contacts, cases were detected using PCR (Delta: 86%; Omicron 87%), antigen test, and PCR (Delta: 13%, Omicron: 12%), while eight cases were reported exclusively with an antigen test, corresponding to 0.6–0.8% of the test-positive contacts. Virus variant information was predominantly identified using PCR variant screening, accounting for 94-95% of the primary cases and contacts with Delta infection and 88-89% of Omicron cases. The remaining cases were identified using whole-genome sequencing.

### Data collection

We limited data collection from 14 December 2021 to 23 January 2022 to avoid bias due to differences in the implementation of the Test-Isolate-Trace-Quarantine regime between Omicron and Delta suspected cases (called TISK in Norway). We included only primary cases who tested positive until 13 January 2022 to ensure that the maximum of ten days of exposure was satisfied for all contacts. The data included only private households of sizes between 2–6 residents. We excluded six primary cases and a total of 36 contacts from the analysis due to previously reported SARS-CoV-2 infections. No household with two primary cases was identified in the data. After applying the inclusion and exclusion criteria, households from 57 of the 64 municipalities in the contact tracing system were included in the final data set.

### Definitions

We defined households comprising persons resident in the same dwelling according to data from Statistics Norway. We defined a household secondary case as an individual registered as close contact of the primary case, living in the same household, and who tested positive for SARS-CoV-2 ≤ 10 days after the test date of the primary case. The vaccination status of the cases and contacts were separated into the following categories: (i) unvaccinated, (ii) one-dose vaccine, (iii) two-dose vaccine, and (iv) three-dose vaccine. To define the vaccine status of the household contacts, we used the test date of the primary case and compared it with the contacts’ vaccination dates. A contact was considered unvaccinated if the primary case’s test date was before the contact’s first dose. A contact was considered as a one-dose vaccine if he/she had received one dose of vaccine (mRNA Vaccines or AstraZeneca vaccine) prior to the test date of his/her primary case. Contacts who had received dose 2 within the last week before the primary case’s test date were also categorized as a one-dose vaccine since there was not enough time to produce immunity after the second dose. A contact was regarded as a two-dose vaccine if he/she had received dose 2 (mRNA) at least 1 week prior to the test date of his/her primary case. A contact was considered a three-dose vaccine if he/she had received dose 3 at least 1 week prior to the test date of his/her primary case. The time interval between the second and the third doses should be ≥120 days. The same schedule was used to define the vaccine status of the primary cases using their test and vaccination dates. There were no cases or contacts with four doses of vaccine in the data set. Further information on definitions used in the study is given in Supplementary Methods, Section S2.

### Statistical analysis

We used binomial regression with a log link to estimate the SAR within the household, comparing Delta with Omicron, assuming test activity and case finding did not vary by variant^18^ as

1 $${Y}_{i}\sim {{{{{\rm{Binomial}}}}}}\left(1,\, p\right)$$

${Y}_{i}$ is the infection status of contact i, where 0: uninfected and 1: infected and *p* is the infection probability of contact *i*. The binomial regression model was stratified on the following covariates: vaccination status of adult primary cases, age group, gender of the contacts, and time since last vaccination in contacts older than 16 years with two-dose vaccine to find the relative risk (RR) between them. For instance, the RR of Omicron infection in males versus females is equivalent to ${{\exp }}({\beta }_{1})$ in the following equation:

2 $${{\log }}\left(p\right)={\beta }_{0}+{\beta }_{1}{X}_{i}$$

where ${X}_{i}$ = 0 if contact *i* who is infected by Omicron is female and ${X}_{i}$ = 1 if the same contact is male.

Vaccine effectiveness (VE) against infection among household contacts 16 years and above was calculated using the following equation

3 $${VE}=1-\left(\frac{{SA}{R}_{{{{{{\rm{vaccinated}}}}}}\,{{{{{\rm{contacts}}}}}}}}{{SA}{R}_{{{{{{\rm{Unvaccinated}}}}}}\,{{{{{\rm{contacts}}}}}}}}\right)$$

Contacts aged 0–15 years were excluded from the VE calculations because, in Norway, children 12–15 years were only eligible for one vaccine dose, while young children 0–11 years were not offered vaccination at the time. The significance level (*α*) was set at 5%. Statistical analyses were performed with Rstudio 1.3.1056.

### Reporting summary

Further information on research design is available in the Nature Research Reporting Summary linked to this article.

## Supplementary information

Author Meta Data

Supplementary Information

Reporting Summary

## Data Availability

A summary of the data is provided in the Supplementary Information. Raw individual data are not publicly available and are protected due to data privacy laws^17^.

## Code availability

The codes are available on request to the first or second authors.
