# Supplementary material for: Increased household transmission and immune escape of the SARS-CoV-2 Omicron compared to Delta variants
Source: Nat Commun. 2022 Sep 29;13:5706. doi: 10.1038/s41467-022-33233-9 (PMC9520116; doi:10.1038/s41467-022-33233-9)
Supplement: Supplementary file 2 — Supplementary Information [file 41467_2022_33233_MOESM2_ESM.pdf]

Supplementary Information: Increased household transmission and immune escape of SARS-CoV-2 Omicron compared to Delta variants

Neda Jalali[1], Hilde K. Brustad[2], Arnoldo Frigessi[2,3], Emily A. MacDonald[1], Hinta Meijerink[1], Siri L. Feruglio[1], Karin M. Nygård[1], Gunnar Rø[1], Elisabeth H. Madslien[1], Birgitte Freiesleben De Blasio[1,2]

[1] Norwegian Institute of Public Health, Oslo, Norway

[2] Oslo Centre for Biostatistics and Epidemiology, University of Oslo, Norway

3 Oslo Centre for Biostatistics and Epidemiology, Oslo University Hospital, Norway

Supplementary Methods

S1: Data sources

We used data from the Norwegian COVID-19 pandemic preparedness registry, Beredt C19 containing individual level information from Norwegian health registries. The following Beredt C19 data sources were used in this study:

- Norwegian vaccination registry (SYSVAK)
  - Vaccination date
  - Vaccine type
- Norwegian Surveillance System for Communicable Diseases (MSIS)
  - Test date
  - Positive test result for SARS-CoV-2, either by RT-PCR or antigen rapid test.
  - Virus variant, detected through whole genome sequencing (WGS) or PCR variant screening.
- Norwegian Population Registry
  - Age
  - Birth
  - Gender
- Municipal contact tracing data (KS Fiks Smittesporing) from 64 Norwegian municipalities in 11 different counties. Only data from 57 municipalities fulfilled the inclusion criteria and were included in the study.
  - Linkage between index cases and their corresponding close contacts, identified through manual contact tracing by trained personnel in the municipalities.
- Statistics Norway (SSB)
  - Household ID key

S2: Definitions

Household primary case: The person in a household that initiated the contact tracing in that household and who has a positive-result test datefor SARS-CoV-2 in the period from 14 December 2021-13 January 2022. The person that initiated the contact tracing is identified by only being registered as an index case in the KS Fiks Smittesporing system and not registered as a contact.

Household member: All individuals registered in the same dwelling, based on Statistics Norway (SSB) household data.

Household contact: Contacts identified by the contact tracing team, registered in the contact tracing system, and matched with Statistics Norway (SSB) to determine that they belong to the same household as the primary case.

Test date: Test date of SARS-CoV-2 infection according to the Norwegian Surveillance System for Communicable Diseases (MSIS), including test results from Norwegian test centers. Individuals who had both an antigen test and a PCR test are registered with the test date of the PCR test.

Household secondary case: An individual registered as a contact of the primary case by the municipal contact tracing teams, living in the same household and who had a positive-result test date within and including ten days after the test date of the primary case.

Household secondary attack rate (SAR): The probability that an infectious person infects a person in the same household during his or her infectious period. We considered the infectious period of the primary cases as ten days from their positive test dates.

Variant identification: The variant was determined by the PCR screening, whole genome sequencing (WGS), or both methods.

S3: Study sample characteristics by age and vaccination status

Table S1: Omicron households: age and vaccination status of primary cases and contacts										
Age group	Unvacc.	%	1-dose	%	2-doses	%	3-doses	%	Total	%
**Primary cases**										
0-15 years	109	71.2	43	28.1	1	0.7	NA	0.0	153	100.0
16+ years	42	8.2	26	5.1	371	72.2	75	14.6	514	100.0
Total	151		69		372		75		667	
**Household contacts**										
0-15 years	278	78.1	78	21.9	NA	0.0	NA	0.0	356	100.0
16+ years	44	4.7	43	4.6	654	69.4	202	21.4	943	100.0
Total	322		121		654		202		1299	

Table S2: Delta households: age and vaccination status of primary cases and contacts										
Age group	Unvacc.	%	1-dose	%	2-doses	%	3-doses	%	Total	%
**Primary cases**										
0-15 years	141	87.6	20	12.4	NA	0.0	NA	0.0	161	100.0
16+ years	61	20.7	15	5.1	204	69.4	14	4.8	294	100.0
Total	202		35		204		14		455	
**Household contacts**										
0-15 years	236	82.2	49	17.1	2	0.7	NA	0.0	287	100.0
16+ years	61	10.5	13	2.2	443	76.0	66	11.3	583	100.0
Total	297		62		445		66		870	

Table S3: Numbers of contacts/infected contacts of primary cases infected with Omicron and Delta variants, stratified by age, vaccination status, gender and time since infection.

Stratification	Omicron			Delta		
	Contacts (N)	Infected Contacts (n)	%	Contacts (N)	Infected Contacts (n)	%
**Overall**	1299	662	51.0	870	315	36.2
**Age of contacts**						
<16 years	356	206	57.9	287	120	41.8
16+ years	943	455	48.3	583	195	33.4
**Age of primary cases**						
<16 years	354	188	53.1	353	123	34.8
16+ years	945	473	50.1	517	192	37.1
**Vacc. status, contacts**						
Unvacc.	322	190	59.0	297	138	46.5
1-dose	121	69	57.0	62	21	33.9
2-doses	654	326	49.8	445	143	32.1
3-doses	202	76	37.6	66	13	19.7
**Vacc. status, primary cases**						
Unvacc.	329	187	56.8	420	158	37.6
1-dose	154	64	41.6	71	28	39.4
2-doses	713	363	50.9	360	127	35.3
3-doses	103	47	45.6	19	2	10.5
**Vacc. Status, contacts 16+ years**						
Unvacc.	44	30	68.2	61	34	55.7
1-dose	43	23	53.5	13	5	38.5
2-doses	654	326	49.8	443	143	32.3
3-doses	202	76	37.6	66	13	19.7
**Vacc. Status, primary cases 16+ years**						
Unvacc.	51	23	45.1	61	29	47.5
1-dose	44	20	45.5	17	8	47.1
2-doses	499	235	47.1	235	70	29.8
3-doses	83	37	44.6	12	1	8.3
**Gender**						
Female	513	259	50.5	294	97	30.0
Male	430	196	45.6	289	98	33.9
**Time since last vacc., contacts w.2-doses 16+ years**						
≤ 6 months	45	20	44.4	46	10	21.7
>6 months	609	306	50.2	397	133	33.5

S4: Sensitivity analysis: using five days between the infected contacts' test dates and their primary cases' test dates

Table S4: five-day secondary attack rate (SAR5) of Omicron and Delta stratified by age and vaccination status			
Stratification	SAR (95% CI)		RR (95% CI)
	Omicron	Delta	Omicron vs Delta (Delta is Ref)
**Overall**	0.46 (0.43 - 0.49)	0.31 (0.28 - 0.35)	**1.47 (1.30 - 1.66)**
**Age of contacts**			
<16 years	0.53 (0.47 - 0.58)	0.37 (0.31 - 0.43)	**1.42 (1.17 - 1.73)**
16+ years	0.43 (0.40 - 0.47)	0.28 (0.245 - 0.32)	**1.53 (1.31 - 1.80)**
**Age of primary cases**			
<16 years	0.46 (0.42 - 0.49)	0.28 (0.23 - 0.34)	**1.65 (1.33 - 2.07)**
16+ years	0.47 (0.41 - 0.53)	0.33 (0.29 - 0.37)	**1.38 (1.19 - 1.61)**
**Vaccine status, contacts**			
Unvaccinated	0.54 (0.48 - 0.60)	0.43 (0.37 - 0.50)	**1.24 (1.04 - 1.49)**
1-dose vaccine	0.53 (0.43 - 0.63)	0.25 (0.143 - 0.37)	**2.16 (1.36 - 3.80)**
2-dose vaccine	0.45 (0.41 - 0.49)	0.26 (0.22 - 0.31)	**1.74 (1.44 - 2.11)**
3-dose vaccine	0.32 (0.26 - 0.39)	0.19 (0.107 - 0.30)	**1.69 (1.02 - 3.11)**
**Vaccine status, primary cases**			
Unvaccinated	0.51 (0.46 - 0.57)	0.32 (0.27 - 0.37)	**1.62 (1.34 - 1.97)**
1-dose vaccine	0.33 (0.26 - 0.42)	0.35 (0.24 - 0.47)	0.96 (0.64 - 1.48)
2-dose vaccine	0.47 (0.43 - 0.51)	0.31 (0.26 - 0.36)	**1.51 (1.26 - 1.81)**
3-dose vaccine	0.40 (0.30 - 0.50)	0.11 (0.02 - 0.29)	**3.76 (1.30 - 21.90)**

SAR: Secondary Attack Rate; CI: Confidence Interval; RR: relative risk, unadjusted

**Table S5: Vaccine efficacy (VE) of Omicron and Delta by contact vacc. status (16+ years) using SAR5**		
Vaccine status	VE % (95% CI)	
	Omicron	Delta
Unvaccinated	Ref	Ref
1-dose vaccine	21 (0 - 51)	32 (0 - 76)
2-dose vaccine	29 (4 - 44)	51 (32 - 63)
3-dose vaccine	50 (28 - 63)	65 (40 - 81)

VE: Vaccine Efficacy; CI: Confidence Interval

**Table S6: Relative risk of Omicron and Delta infection stratified by different covariates using SAR5**		
RR within variant	RR (95% CI)	
	Omicron	Delta
**Primary case vaccination status (16+)**		
Unvaccinated	Ref	Ref
1-dose vaccine	0.96 (0.55 - 1.65)	1.04 (0.46 - 1.92)
2-dose vaccine	1.09 (0.78 - 1.66)	**0.62 (0.42 - 0.95)**
3-dose vaccine	1.06 (0.69 - 1.71)	**0.20 (0.01 - 0.82)**
**Age of contacts**		
<16 years	1.41 (0.99 - 2.23)	1.30 (0.78 - 2.64)
16-44 years	1.29 (0.90 - 2.03)	0.96 (0.57 - 1.97)
45-64 years	1.04 (0.72 - 1.65)	1.04 (0.60 - 2.16)
65+ years	Ref	Ref
**Gender of contacts, 16+ years**		
Female	Ref	Ref
Male	0.91 (0.77 - 1.06)	1.01 (0.77 - 1.34)
**Time since last vaccination, 2-dose vacc. contacts 16+ years**		
≤ 6 months	0.98 (0.66 - 1.33)	0.53 (0.22 - 1.03)
>6 months	Ref	Ref

RR: Relative Risk, unadjusted; CI: Confidence Interval
