# Supplementary material for: Increased household transmission and immune escape of the SARS-CoV-2 Omicron compared to Delta variants
Source: Nat Commun. 2022 Sep 29;13:5706. doi: 10.1038/s41467-022-33233-9 (PMC9520116; doi:10.1038/s41467-022-33233-9)
Supplement: Supplementary file 3 — Reporting Summary [file 41467_2022_33233_MOESM3_ESM.pdf]

nature portfolio

Corresponding author(s): Birgitte Freiesleben de Blasio

Last updated by author(s): Sep 6, 2022

Reporting Summary

Nature Portfolio wishes to improve the reproducibility of the work that we publish. This form provides structure for consistency and transparency in reporting. For further information on Nature Portfolio policies, see our Editorial Policies and the Editorial Policy Checklist.

Statistics

For all statistical analyses, confirm that the following items are present in the figure legend, table legend, main text, or Methods section.

n/a	Confirmed	
☐	☒	The exact sample size (*n*) for each experimental group/condition, given as a discrete number and unit of measurement
☒	☐	A statement on whether measurements were taken from distinct samples or whether the same sample was measured repeatedly
☒	☐	The statistical test(s) used AND whether they are one- or two-sided<br>*Only common tests should be described solely by name; describe more complex techniques in the Methods section.*
☐	☒	A description of all covariates tested
☒	☐	A description of any assumptions or corrections, such as tests of normality and adjustment for multiple comparisons
☐	☒	A full description of the statistical parameters including central tendency (e.g. means) or other basic estimates (e.g. regression coefficient) AND variation (e.g. standard deviation) or associated estimates of uncertainty (e.g. confidence intervals)
☒	☐	For null hypothesis testing, the test statistic (e.g. *F*, *t*, *r*) with confidence intervals, effect sizes, degrees of freedom and *P* value noted<br>*Give P values as exact values whenever suitable.*
☒	☐	For Bayesian analysis, information on the choice of priors and Markov chain Monte Carlo settings
☒	☐	For hierarchical and complex designs, identification of the appropriate level for tests and full reporting of outcomes
☒	☐	Estimates of effect sizes (e.g. Cohen's *d*, Pearson's *r*), indicating how they were calculated

Our web collection on statistics for biologists contains articles on many of the points above.

Software and code

Policy information about availability of computer code

Data collection	Not relevant. No software was used for data collection
Data analysis	Statistical analyses were performed with RStudio version 1.3.1056. The source code does not contain custom algorithms; it can be shared at personnel request to the first or second authors.

For manuscripts utilizing custom algorithms or software that are central to the research but not yet described in published literature, software must be made available to editors and reviewers. We strongly encourage code deposition in a community repository (e.g. GitHub). See the Nature Portfolio guidelines for submitting code & software for further information.

Data

Policy information about availability of data

All manuscripts must include a data availability statement. This statement should provide the following information, where applicable:

- Accession codes, unique identifiers, or web links for publicly available datasets
- A description of any restrictions on data availability
- For clinical datasets or third party data, please ensure that the statement adheres to our policy

A summary of the data is provided in the Supplementary Methods. Raw individual data are from the emergency preparedness registry Beredt C19 is not publicly available and are protected due to data privacy laws. Requests for data from Beredt C19 should be forwarded to Beredt C19; see https://www.fhi.no/en/id/

infectious-diseases/coronavirus/emergency-preparedness-register-for-covid-19/ for further information. The time frame for a response may vary depending on capacity. Requests for data will be handled in accordance with Norwegian law and the GDPR.

Human research participants

Policy information about studies involving human research participants and Sex and Gender in Research.

Reporting on sex and gender	*Use the terms sex (biological attribute) and gender (shaped by social and cultural circumstances) carefully in order to avoid confusing both terms. Indicate if findings apply to only one sex or gender; describe whether sex and gender were considered in study design whether sex and/or gender was determined based on self-reporting or assigned and methods used. Provide in the source data disaggregated sex and gender data where this information has been collected, and consent has been obtained for sharing of individual-level data; provide overall numbers in this Reporting Summary. Please state if this information has not been collected. Report sex- and gender-based analyses where performed, justify reasons for lack of sex- and gender-based analysis.*
Population characteristics	*Describe the covariate-relevant population characteristics of the human research participants (e.g. age, genotypic information, past and current diagnosis and treatment categories). If you filled out the behavioural & social sciences study design questions and have nothing to add here, write "See above."*
Recruitment	*Describe how participants were recruited. Outline any potential self-selection bias or other biases that may be present and how these are likely to impact results.*
Ethics oversight	*Identify the organization(s) that approved the study protocol.*

Note that full information on the approval of the study protocol must also be provided in the manuscript.

Field-specific reporting

Please select the one below that is the best fit for your research. If you are not sure, read the appropriate sections before making your selection.

☒ Life sciences ☐ Behavioural & social sciences ☐ Ecological, evolutionary & environmental sciences

For a reference copy of the document with all sections, see nature.com/documents/nr-reporting-summary-flat.pdf

Life sciences study design

All studies must disclose on these points even when the disclosure is negative.

Sample size	No sample size calculations were performed. We used all available information from the Norwegian emergency response registry Beredt C19. Some exclusion criteria were applied, see below.
Data exclusions	We limited the study to include households of sizes 2-6 to exclude institutions, including care facilities and nursing homes. Six primary cases and 37 contacts (including contacts of the six primary cases) were excluded due to previously registered infections. We limited the data collection period to 14 December 2021 - 23 January 2022 where the Test-Trace-Quarantine-Isolation regime in Norway was unchanged to limit bias from control policies. Eligible primary cases were registered with infection between 14 December 2021 and 13 January 2022 to ensure an exposure period of ten days for all contacts.
Replication	Not relevant. The study is time-sensitive, limited to the period December 2021 - January 2022, where the dominance of the Delta variant in Norway was taken over by the Omicron variant, and the Test-Trace-Quarantine-Isolation regime did not change.
Randomization	Not relevant. The study analyses previously collected data during contact tracing individually merged with data from Norwegian registries.
Blinding	Not relevant. The study analyses previously collected data during contact tracing individually merged with data from Norwegian registries.

Reporting for specific materials, systems and methods

We require information from authors about some types of materials, experimental systems and methods used in many studies. Here, indicate whether each material, system or method listed is relevant to your study. If you are not sure if a list item applies to your research, read the appropriate section before selecting a response.

Materials & experimental systems

n/a	Involved in the study
☒	☐ Antibodies
☒	☐ Eukaryotic cell lines
☒	☐ Palaeontology and archaeology
☒	☐ Animals and other organisms
☒	☐ Clinical data
☒	☐ Dual use research of concern

Methods

n/a	Involved in the study
☒	☐ ChIP-seq
☒	☐ Flow cytometry
☒	☐ MRI-based neuroimaging
